# Antibiotic resistance in mucosal bacteria from high Arctic migratory salmonids

**DOI:** 10.1111/1758-2229.12975

**Published:** 2021-06-09

**Authors:** Kristy Moniz, Virginia K. Walker, Vishal Shah

**Affiliations:** ^1^ Department of Biology and School of Environmental Studies Queen's University Kingston Ontario K7L 3N6 Canada; ^2^ College of the Sciences and Mathematics West Chester University West Chester Pennsylvania USA

## Abstract

Two related salmonids, Arctic char (*Salvelinus alpinus*) and lake whitefish (*Coregonus clupeaformis*) sampled from the high Arctic region of Nunavut, Canada are anadromous fish, migrating annually from the same ice‐covered freshwater waterbodies to spend summers in the marine waters of the Arctic Ocean. Microbiota associated with the skin‐associated mucus undergo community change coincident with migration, and irrespective of this turnover, antibiotic resistance was detected in mixed bacterial cultures initiated with mucus samples. Although as expected most bacteria were unculturable, however, 5/7 isolates showed susceptibility to a panel of five common antibiotics. The fish were sampled under severe conditions and at remote locations far from human habitation. Regardless, two isolates, ‘*Carnobacterium maltaromaticum* sm‐2’ and ‘*Arthrobacter citreus* sm’, showed multi‐resistance to two or more antibiotics including ampicillin and streptomycin indicating multiple resistance genes. It is unknown if these fish bacteria have ‘natural’ resistance phenotypes or if resistance has been acquired. As result of these observations, we urge long‐term monitoring of drug‐resistant bacteria in the region and caution the assumption of a lack of drug‐resistant organisms even in such extreme environments.

## Introduction

Members of the salmonid family of teleost fish, Arctic char (*Salvelinus alpinus*) and lake whitefish (*Coregonus clupeaformis*), were sampled from the high Arctic. These fish spend summers feeding in the rich waters of the Arctic Ocean but must swim up rivers to inland lakes in order to escape sub‐zero temperatures during sea ice formation in the autumn, with these two related species migrating at the same time and on the same routes (Moore *et al*., 2017; Hamilton *et al*., 2019; Element *et al*., 2020). Such migratory behaviour, known as anadromy, is a challenge to fish health with seasonal changes in salinity forcing osmoregulatory changes involving adaptations to the gills, alimentary tract, epithelium, and kidney function (McCormick, 2012; Kononova *et al*., 2019). Also critical during migration is the conservation of the fish immune system associated with the mucosal surfaces of the skin (Llewellyn *et al*., 2014). This acts as the first line of defence against pathogens and is fortified with defence cells and antibacterial peptides, with the mucus also hosting commensal bacteria that show seasonal salinity‐associated turnover (Lokesh and Kiron, 2016; Hamilton *et al*., 2019; Element *et al*., 2020). These bacteria found in the mucus likely provide protection against pathogenic bacteria and the risk of dysbiosis in migrating salmonids. Here, we hypothesized that the microbiota employ strategies to ensure their persistence in skin‐associated communities. Initially, we have tested our hypothesis by the characterization of skin mucous isolates for antibiotic resistance.

Of the many discoveries of 20th century that have impacted the life span and quality of mankind, antibiotic discovery has been one of the major advancements in the field of medicine (Gould and Bal, 2013). The use of antibiotics has contributed to the 1.4‐fold extension of human life spans over the last 100 years in western nations (Rossolini *et al*., 2014; Ventola, 2015), with non‐medical applications in agricultural practise including use as a growth enhancers in livestock production (Martinez, 2009; Manyi‐Loh *et al*., 2018). Although the widespread use of antibiotics has resulted in increased prevalence of antibiotic‐resistant microorganisms in the environment, it is also possible that commensal bacteria could naturally harbour antibiotic‐resistant phenotypes related to their function and persistence, or that resistome acquisition could be beneficial for the probiotic continuance.

In this regard, it is acknowledged that ‘natural’ antibiotic‐resistance distant from human habitation is not widespread although antibiotic resistance genes have been occasionally recovered from glaciers in five continents, and ampicillin resistance was reported in polar bear microbiota, and in freshwater samples collected well away from human activities in Antarctica (Glad *et al*., 2010; Segawa *et al*., 2013; Jara *et al*., 2020). In the current study, we have investigated whether antibiotic‐resistant bacteria are present in the mucosal community of anadromous salmonids caught in the lower Northwest Passage of the Kitikmeot region of Nunavut, Canada. Fishing sites were all located about 130 km from Gjoa Haven Nunavut, a hamlet of 1300 people with neither agricultural activities nor hospital facilities. Our goal was to determine whether antibiotic resistance, possibly acquired naturally from this relatively ‘clean’ environment was present in mucus‐associating microbiota.

## Results and discussion

Mucosal samples were cultured as a mixed consortium under the conditions described including fish caught at three different sites in either the spring or fall fishing seasons, during migration either to the sea or to lakes, respectively. Not all the samples could be cultured (about 13/24, with 10 of the better growing cultures retained). As is well known, only a fraction of a percent of environmental isolates can be successfully cultured (e.g. Amann *et al*., 1995; Schleifer, 2004; Walker *et al*., 2006). Mucosal samples studied here are no exception and we were able to culture only a small fraction of the bacteria identified as part of the microbiota (Hamilton *et al*. 2019). Only three genera with a final abundance ≥1%, in the mixed cultures, including *Carnobacterium*, *Psychrobacter* and *Granulicatella* were identified (Supporting Information Table S1), with other bacteria at <1%. In all the mucosa samples analysed, *Carnobacterium* and *Psychrobacter* combined for over 95% of the cultured microbial community (Supporting Information Table S1). *Carnobacterium* is a well‐known cold‐water bacterium, *Psychrobacter* has been recovered from polar marine environments and ice cores, and *Granulicatella* has been associated with biofilms and marine organisms (Collins *et al*., 2010; Iskandar *et al*., 2017; Gong *et al*., 2020).

After antibiotic exposure, variation in community composition of the mixed cultures was observed (Supporting Information Table S1). Strikingly, when comparing the percentage of each of the bacteria that made up the cultured community assessed in the presence or absence of each antibiotic, all the bacteria at the genera level are resistant to at least one antibiotic tested (Table 1; Supporting Information Table S1). For example, *Carnobacterium* species were resistant to kanamycin and ampicillin but susceptible to tetracyline. Considerable phenotypic variation was seen when these consortia were cultured in presence of streptomycin and chloramphenicol. We interpret this to suggest that resistance to these antibiotics was not widespread, with drug‐resistant bacteria variably present in the salmonids. *Psychrobacter* species were susceptible to a single antibiotic, kanamycin, and resistant to tetracycline. Further, this organism was resistant in the mucosa samples of some fish and sensitive in others (variable resistance) to ampicillin, streptomycin, and chloramphenicol. In contrast, *Granulicatella* species were more susceptible to the addition of antibiotics compared to other tested bacteria but showed some resistance to three of the antibiotics tested. Overall, bacteria from mixed cultures showed resistance to ampicillin and streptomycin.

**Table 1:** Qualitative assessment of antibiotic sensitivity of the microorganisms present in the mucosa of *S. alpinus* when grown as a mixed community in presence of antibiotics, with quantitative data shown in the Supplementary Table S1, and with the following qualitative assessments: Resistant, % composition of the organisms is higher in presence of antibiotic when compared to control in all the mucosa samples analysed; Sensitive, % composition of the organism is lower in presence of antibiotic when compared to control in all the mucosa samples analysed; Variable, % composition of the organisms is higher in some mucosa samples and lower in other samples in presence of antibiotics when compared to control.

Antibiotic	*Granulicatella*	*Psychrobacter*	*Carnobacterium*
Ampicillin	Resistant	Variable	Resistant
Chloramphenicol	Sensitive	Variable	Variable
Kanamycin	Variable	Sensitive	Resistant
Streptomycin	Variable	Variable	Variable
Tetracycline	Sensitive	Resistant	Sensitive

Attempts to obtain pure plate cultures from the initial mixed mucosal liquid cultures resulted in seven independent isolates with a single isolate chosen from each mixed culture (Table 2). Curiously, even though *Granulicatella* was one of the three most abundant genera in the mixed liquid culture, subsequently no single colonies belonging to this genus could be isolated. We speculate that growth of these bacteria was dependent on other species in the consortium. Consistent with this interpretation, it has previously been reported that when plated on solid medium, *Granulicatella* appears as satellite colonies around other organisms such as *Staphylococcus aureus* or *S. epidermidis* that provide nutrients for growth (Koh *et al*., 2014). Sequencing of the 16S rRNA gene fragments from each colony identified the seven isolates to their closest relatives and this has been shown along with metadata associated with their original fish hosts (Table 2). Each isolate was tested for antibiotic resistance and comparison of the MIC_50_ values demonstrated that in contrast to the mixed cultures, five of the seven isolates were susceptible to all the antibiotics tested (MIC_50_ values < 1 μg μl^−1^; Table 2). Notably, however, the remaining two isolates, both derived from *S. alpinus* mucus, showed multi‐drug resistance; ‘*Carnobacterium maltaromaticum* sm‐2’ (the second *Carnobacterium* salmonoid mucus isolate recovered) showed resistance to three of the antibiotics tested, ampicillin, kanamycin and streptomycin (MIC_50_ values > 1 μg μl^−1^), and ‘*Arthrobacter citreus* sm’, was resistant to ampicillin and streptomycin (Table 2). The observed multi‐resistant phenotype for both ampicillin and streptomycin in these two isolates was consistent with the liquid assay for the mixed cultures (Table 1). Never‐the‐less, neither of these Gram‐positive multidrug resistant bacteria are salmonid pathogens of concern. Previous research has shown that the organisms may be of positive benefit to the fish, with *C. maltaromaticum* and *A. citreus* showing antibacterial and antifungal activity, respectively (Kerr, 1999; Ringø, 2008).

**Table 2:** Characterization of Arctic salmonid skin‐associated mucosal isolates, indicating the closest accession numbers for the 16S rRNA sequences with species shown if identity was ≥97% but in quotes since the identification was based on sequence identity only and with the addition of a sm (salmonid mucosa) subscript, the fish identification (ID) number and salmonid species, collection time, the distance from the hamlet, as well as antibiotic resistance including the Minimal Inhibitory Concentration (MIC_50_) values of pure cultures of the respective isolates in the presence of the antibiotics listed.

Isolates	*Arthrobacter citreus* sm	*Carnobacterium maltaromaticum* sm‐1	*Carnobacterium maltaromaticum* sm‐2	*Psychrobacter alimentarius* sm	*Psychrobacter cibarius* sm	*Rhodococcus sp*. sm	*Sphingomonas sp* sm
Closest Accession	NR026188.1	NR044710	NR044710.2	NR025798.1	NR043057.1	NR116275.1	NR042129
Identity (%)	99.4	98.2	98.1	99.9	99.5	99.6	99.1
km from hamlet	130	130	135	135	120	130	130
Water type	Brackish	Brackish	Brackish	Brackish	Fresh #2106/1	Brackish	Brackish
Fish ID/site	#885/7	#803/7	#886/6	#880/6	Dec. 2016	#457/7	#824/7
Fishing date	Sept. 2016	Sept. 2016	Sept. 2016	Sept. 2016	*Salvelinus*	Sept. 2018	Sept. 2016
Salmonid type	*Salvelinus*	*Salvelinus*	*Salvelinus*	*Salvelinus*		*Coregonus*	*Salvelinus*
Antibiotic Resistance:	Yes	No	Yes	No	No	No	No
Ampicillin					0.48		
MIC_50_ (μg/μl)	2.4	0.096	300	0.48		0.48	0.48
Kanamycin					<7.7 × 10^−5^		
MIC_50_ (μg/μl)	0.0096	<7.7 × 10^−5^	6	0.0096		<7.7 × 10^−5^	<7.7 × 10^−5^
Tetracycline					0.012		
MIC_50_ (μg/μl)	1.9 × 10^−5^	1.9 × 10^−5^	0.00048	0.012		0.012	0.012
Streptomycin					0.0096		
MIC_50_ (μg/μl)	6	0.24	30	0.24		<7.7 × 10^−6^	0.24
Chloramphenicol					0.024		
MIC_50_ (μg/μl)	0.0048	0.0048	0.6	0.0048		0.024	0.0048

We acknowledge that the number of antibiotic‐resistant organisms in the mucosa of Arctic salmonids could be larger than that observed in our study. Fish were caught in remote Arctic areas and sometimes under the ice, with freezing of some fish occurring as soon as the nets were pulled above water into sub‐zero air temperatures. Thus, freezing was the only practical way to return all the samples from distant fishing to the laboratory, which also ensured that there was no spoilage. It is known, however, that bacterial communities in the mucosa are protected by the presence of fish mucus, which contains protein and mucins, the latter which are known to preserve bacteria for years at sub‐zero temperatures (Hubálek, 2003). Notwithstanding this consideration, there is the possibility of decreased bacterial viability due to freezing. In addition, assays for antibiotic resistance in microbial consortia were conducted at concentrations ranging from 5 μg μl^−1^ to 100 μg μl^−1^. We allow that by testing at lower antibiotic concentrations and transport at low, but non‐freezing temperatures, more bacteria showing low levels of resistance might have been recovered.

It is none‐the‐less clear from the observed results that some bacteria found in the mucus of anadromous Arctic salmonids are resistant to antibiotics. Whether this is an ancient attribute, evolved as part of a commensal strategy to help protect host fish from pathogens by preserving beneficial bacteria is uncertain. Indeed, it has been reported that some antibiotic resistance is ‘intrinsic’ to bacteria from tilapia fish internal organs (Wamala *et al*., 2018). Certainly, *C. maltaromaticum* effectively produces bacteriocins that inhibit disease‐producing Gram‐positive pathogenic bacteria in the gut of salmonids (Ringø, 2008), but the nature of these is unknown, and the mechanism whereby the bacteriocin‐producers remain viable remains undiscovered. Alternatively, the acquisition of antibiotic resistance could be more recent, coincident with the rise of the use of these therapies in health and agriculture. Even though individual Arctic salmonids may live for several decades, seasonal ‘turnover’ would allow fresh colonization twice yearly. Given the multi‐drug‐resistant phenotype of two of the isolates, this could suggest that gene transfer of resistomes may occur even in the high Arctic. Given that the fishing sites were at least 120 km from the closest human habitation consisting of a small hamlet without a hospital or agricultural activities (Table 2), we speculate that such resistomes may be derived from some of the more than 100 different migratory bird species in this region (Latour *et al*., 2008). Migratory birds could likely bring bacteria and as well as antibiotic resistance genes from the south to their breeding grounds in the most remote part of the polar regions (Sjölund *et al*., 2008; Hernández and González‐Acuña, 2016). Once the genes are introduced in the community, the genetic plasticity of the microbial community could enable resistance genes to move quickly throughout different environmental bacterial populations and communities (Watts *et al*., 2017). If this is the case, climate change and the consequent increasing economic activities in the region may provide selective pressure for the increase of antibiotic resistance in the Arctic. We have already urged monitoring of anthropogenic contaminants in these fish (Walker *et al*., 2020), and we suggest that it would be prudent to on‐going monitoring of the prevalence of drug‐resistant bacteria in the region, and in‐particular for human and animal pathogens.

## Supporting information


**Appendix S1**: Supporting Information

**Table S1** Genera comparison of mucosa microbial community cultured in 10% TSB‐ASW media for individual fish samples in absence (C, Control) and presence of antibiotics (A, Ampicilin; K, Kanamycin; T, Tetracycline; S, Streptomycin; Ch, Chloramphenicol).
