# Supplementary material for: Antibiotic resistance in mucosal bacteria from high Arctic migratory salmonids
Source: Environ Microbiol Rep. 2021 Jun 9;14(3):385–90. doi: 10.1111/1758-2229.12975 (PMC9292178; doi:10.1111/1758-2229.12975)
Supplement: Supplementary file 1 — Appendix S1: Supporting Information [file EMI4-14-385-s001.docx]

**METHODS**

**Sampling, Sequencing and Assessment of Antibiotic Resistance in Communities:**

Salmonid adults were caught in nets in remote areas of the high Arctic at traditional fishing sites and with the assistance of the Hunters and Trappers Association of Gjoa Haven, Nunavut. Each fish was assigned a unique barcode to facilitate the tracking of samples during transport and storage (Wu *et al*., 2020). This barcode can be used to access the metadata on each fish including the barcode identifier, species, water salinity at the net site among other information, as posted on the Polar Data Catalogue (CCIN 12992, file named “Updated Master data for PDC”) Mucosal microbiome samples were aseptically collected onto sterile swabs as soon as each fish was landed using the protocol described in earlier studies (Kuppulakshi *et al.,* 2008; Hamilton *et al*., 2019, Kumari *et al.*, 2019). All samples were frozen at -20 °C and shipped on frozen freezer packs to the laboratory. Culturing the community consortia was accomplished by recovering 100 μL of the mucus from the frozen swabs and transfer to a sterile conical tube containing 10 mL of sterile 10% tryptic soy broth made with 40% artificial sea water, designated 10% TSB-ASW consisting of 23.4 g NaCl, 4.9 g MgSO_4_*7H_2_O, 1.1 g CaCl_2_*2H_2_O, 0.2 g KBr, 0.75 g KCl, and 4.1 g MgCl_2_*6H_2_O in 1 L of distilled water (Leifson, 1963; Pratt, 1963; Harrison and Berges, 2005; Komives *et al*., 2005). The cultures were then incubated at 12 °C for 3 days.

Antibiotic sensitivity of the cultured microbiota consortium was tested using by adding 100 μL of the consortium to 5 mL of 10% TSB-ASW supplemented with antibiotics (Fisher Scientific, Ontario, Canada). Experimental parameters included control tubes with no antibiotics and experimental tubes containing ampicillin (100 μg/μL), streptomycin (50 μg/μL), tetracycline (5 μg/μL), chloramphenicol (25 μg/μL) and kanamycin (50 μg/μL). Tubes were incubated at 12 °C for 3 days and then centrifuged for 2 min at 13,000 x g. DNA was extracted from the pellet using MoBio UltraClean Tissue and Cells DNA Isolation kit (Qiagen, Montreal QC) using the manufacturer’s standard protocol with a final elution using 50 μL of sterile water. The DNA was assessed for quality and quantity by agar gel electrophoresis and NanoDrop One spectrophotometry (ThermoFisher Scientific, Ottawa, ON) analysis, respectively, before shipment to Molecular Research DNA (Shallowwater, Texas), for Illumina analysis. All protocols including 16S rRNA gene sequence amplification and appropriate primers, Illumina miSeq protocols, sequence analysis and pipelines were performed as described previously in detail (Dowd *et al*., 2008, Chiodini *et al.*, 2015, Garcia-Mazcorro *et al*., 2017). In brief, PCR amplification of the V3-V4 region of the 16S rRNA gene was carried out using 515F and 806R primers. Samples were processed by using the NexteraXT Library Preparation Kit (Illumina) in accordance with the manufacturer’s protocol for 16S metagenomic sequencing. Amplicons were sequenced on the MiSeq platform (Illumina, San Diego, CA). Once the sequences were joined, sequences <150 bp and sequences with ambiguous base calls were removed. Sequences were quality filtered using a maximum expected error threshold of 1.0 and dereplicated. The dereplicated or unique sequences are denoised; unique sequences identified with sequencing and/or PCR point errors and removed, followed by chimera removal, thereby providing a denoised sequence. Final denoised sequences were taxonomically classified using BLASTn against a curated database derived from NCBI. The dataset is available at the NCBI under BioProject accession number PRJNA604977.

Comparison to the control samples allowed for the identification of a particular phenotype, either sensitive or resistant to a given antibiotic, when present as part of the microbiota consortium. This dataset is available at the NCBI under BioProject accession number PRJNA701660

**Identification and Assessment of Resistance in Individual Isolates:**

To recover individual bacterial isolates from the consortium, serial dilution and plating methods were employed on 10% TSB-ASW medium and plates (supplemented with 1.5 % agar). After incubation at 12 °C for 3-5 days, individual distinct colonies were selected from 10% TSB-ASW agar plates. The purity of the cultures was confirmed by sequentially streaking and re-culturing multiple times. To identify individual isolates, polymerase chain reaction (PCR) was performed on single colonies using the universal 16S rRNA gene primers 8F (Sebastião *et al*., 2015) and 1406R (Wald *et al*., 2015) to amplify the V1 – V9 portion of the 16S rRNA gene sequence. Purified PCR products were sent to the [Plateforme de Séquençage et de Génotypage des Génomes (Université Laval, QC) for Sanger sequencing, with the sequences subsequently compared using the](http://www.sequences.crchul.ulaval.ca/) Blastn database for identification to the closest matches. These sequences are available in the Polar Data Catalogue (accession CCIN 12992).

Resistance phenotypes to a specific antibiotic was determined using Minimal Inhibitory Concentration of each antibiotic required to decrease the growth of the organism by 50% compared to that of controls (MIC_50_). MIC_50_ values were obtained using 5-fold serial dilution in a microtiter plate. All antibiotic resistance experiments were performed in triplicate or more.

**REFERENCES**

Chiodini, R.J., Dowd, S.E., Chamberlin, W.M., Galandiuk, S., Davis, B., and Glassing, A. (2015) Microbial population differentials between mucosal and submucosal intestinal tissues in advanced Crohn's disease of the ileum. *PLoS one* *10*: e0134382.

Dowd, S.E., Callaway, T.R., Wolcott, R.D., Sun, Y., McKeehan, T., Hagevoort, R.G., and Edrington, T.S. (2008) Evaluation of the bacterial diversity in the feces of cattle using 16S rDNA bacterial tag‐encoded FLX amplicon pyrosequencing (bTEFAP). *BMC Microbiol* *8*: 125.

Garcia‐Mazcorro, J.F., Castillo‐Carranza, S.A., Guard, B., Gomez‐Vazquez, J.P., Dowd, S.E., and Brigthsmith, D.J. (2017) Comprehensive molecular characterization of bacterial communities in feces of pet birds using 16S marker sequencing. *Microb Ecol* *73*: 224–235.

Harrison, P.J., and Berges, J.A. (2005) Marine culture medium. In *Algal Culturing Techniques*. Andersen, R.A. (ed). San Diego: Academic Press, pp. 21–33.

Komives, C.F., Cheung, L.Y.Y., Pluschkell, S.B., and Flickinger, M.C. (2005) Growth of Bacillus methanolicus in seawater‐based media. *J Ind Microbiol Biotechnol* *32*: 61–66.

Kumari, S., Tyor, A.K., and Bhatnagar, A. (2019) Evaluation of the antibacterial activity of skin mucus of three carp species. *Int Aquat Res* *11*: 225–239.

Kuppulakshmi, C., Prakash, M., Gunasekaran, G., Manimegalai, G., and Sarojini, S. (2008) Antibacterial properties of fish mucus from Channa punctatus and Cirrhinus mrigala. *Eur Rev Med Pharmacol Sci* *12*: 149–153.

Leifson, E. (1963) Determination of carbohydrate metabolism of marine bacteria. *J Bacteriol* *85*: 1183–1184. <https://doi.org/10.1128/JB.85.5.1183‐1184.1963>.

Pratt, D. (1963) Specificity of the solute requirement by marine bacteria on primary isolation from sea‐water. *Nature* *199*: 1308.

Sebastião, F., Furlan, L., Hashimoto, D., and Pilarski, F. (2015) Identification of bacterial fish pathogens in Brazil by direct colony PCR and 16S rRNA gene sequencing. *Adv Microbiol* *5*: 409–424. <https://doi.org/10.4236/aim.2015.56042>.

Seveno, N., Smalla, K., van Elsas, J.D., Collard, J.‐M., Karagouni, A., Kallifidas, D., and Wellington, E. (2002) Occurrence and reservoirs of antibiotic resistance genes in the environment. *Rev Med Microbiol* *13*: 15–27.

Wald, J., Hroudova, M., Jansa, J., Vrchotova, B., Macek, T., and Uhlik, O. (2015) Pseudomonads rule degradation of polyaromatic hydrocarbons in aerated sediment. *Front Microbiol* *6*: 1268.

Walke, J.B., Becker, M.H., Hughey, M.C., Swartwout, M.C., Jensen, R.V., and Belden, L.K. (2015) Most of the dominant members of amphibian skin bacterial communities can be readily cultured. *Appl Environ Microbiol* *81*: 6589–6600. <https://doi.org/10.1128/AEM.01486‐15>.

Wu, Y., Lougheed, D.R., Lougheed, S.C., Moniz, K., Walker, V.K., and Colautti, R.I. (2020) baRcodeR: an open‐source R package for sample labelling. *Methods Ecol Evol* *11*: 980–985.
